# Lipid‐based regulators of immunity

**DOI:** 10.1002/btm2.10288

**Published:** 2021-12-31

**Authors:** Wade T. Johnson, Nicholas C. Dorn, Dora A. Ogbonna, Nunzio Bottini, Nisarg J. Shah

**Affiliations:** ^1^ Department of Nanoengineering University of California, San Diego La Jolla California USA; ^2^ Chemical Engineering Program University of California, San Diego La Jolla California USA; ^3^ Division of Rheumatology, Allergy and Immunology, Department of Medicine University of California, San Diego La Jolla California USA; ^4^ Program in Immunology University of California, San Diego La Jolla California USA

**Keywords:** adjuvants, immunomodulation, lipids, polyunsaturated fatty acids, short‐chain fatty acids

## Abstract

Lipids constitute a diverse class of molecular regulators with ubiquitous physiological roles in sustaining life. These carbon‐rich compounds are primarily sourced from exogenous sources and may be used directly as structural cellular building blocks or as a substrate for generating signaling mediators to regulate cell behavior. In both of these roles, lipids play a key role in both immune activation and suppression, leading to inflammation and resolution, respectively. The simple yet elegant structural properties of lipids encompassing size, hydrophobicity, and molecular weight enable unique biodistribution profiles that facilitate preferential accumulation in target tissues to modulate relevant immune cell subsets. Thus, the structural and functional properties of lipids can be leveraged to generate new materials as pharmacological agents for potently modulating the immune system. Here, we discuss the properties of three classes of lipids: polyunsaturated fatty acids, short‐chain fatty acids, and lipid adjuvants. We describe their immunoregulatory functions in modulating disease pathogenesis in preclinical models and in human clinical trials. We conclude with an outlook on harnessing the diverse and potent immune modulating properties of lipids for immunoregulation.

## 1 INTRODUCTION

Lipids, colloquially called fats, are nonpolar hydrocarbons that have a pleiotropic role in modulating physiological functions, including in energy storage, maintaining cell membrane integrity, and cell signaling.^1^ Fatty acids (FAs) are a major class of lipids characterized by a hydrocarbon chain functionalized with a carboxylic acid. FAs are primarily sourced from exogenous sources, such as through dietary essential fatty acids (EFAs) and have been demonstrated to influence the immune response through cell signaling that directly and indirectly activate or suppress cells of both the innate (e.g., neutrophils) and adaptive (e.g., T cells and B cells) immune system. Some FAs are associated with inflammatory diseases and may serve as biomarkers for assessing progression, such as the pro‐inflammatory metabolites of FA called eicosanoids, for chronic conditions such as rheumatoid arthritis,^2^ asthma,^3^ and inflammatory bowel disease (IBD).^4^

In humans, the two principal immunoregulatory FAs are polyunsaturated fatty acids (PUFAs) and short‐chain fatty acids (SCFAs), which together constitute about 15% of the total FAs content, with the remainder comprised of monounsaturated and saturated FAs.^5^ PUFAs are characterized by multiple unsaturations (double bonds) in the lipid tail and follow a “ω‐x” nomenclature, where “x” refers to the carbon atom with the first unsaturation on the aliphatic chain from the methyl terminus. The key structural difference between ω‐3 and ω‐6 PUFAs is in the location of a carbon double bond. This seemingly innocuous change has a significant effect on their metabolic derivatives (eicosanoids) which directly modulate immune cells. SCFAs are small molecule metabolites, comprising less than six carbon atoms with no unsaturations and are derived from the breakdown of complex carbohydrates by gut microbiota.^6^ These products of bacterial fermentation follow the standard IUPAC naming system for carboxylic acids (e.g., propionate [three carbon atoms], butyrate [four carbon atoms]). By activating G‐protein‐coupled cell membrane receptors (GPCRs) and inhibiting histone deacetylase (HDAC), SCFAs can modulate the activity of regulatory immune cells. Outside their structural role in maintaining cell membrane fluidity, extracellular PUFAs are found in tissue microenvironments and are relatively enriched as a fraction of total FA in the brain, heart, and blood plasma.^5^ SCFAs diffuse from the gut lumen, which is the primary site for their biosynthesis, via a strong biological gradient and can partition in tissues throughout the body. At homeostasis, SCFA concentration in tissues outside the intestine is negligible, suggesting that SCFAs primarily affect cells associated with the lower gastrointestinal tract.^6^

**FIGURE 1:**
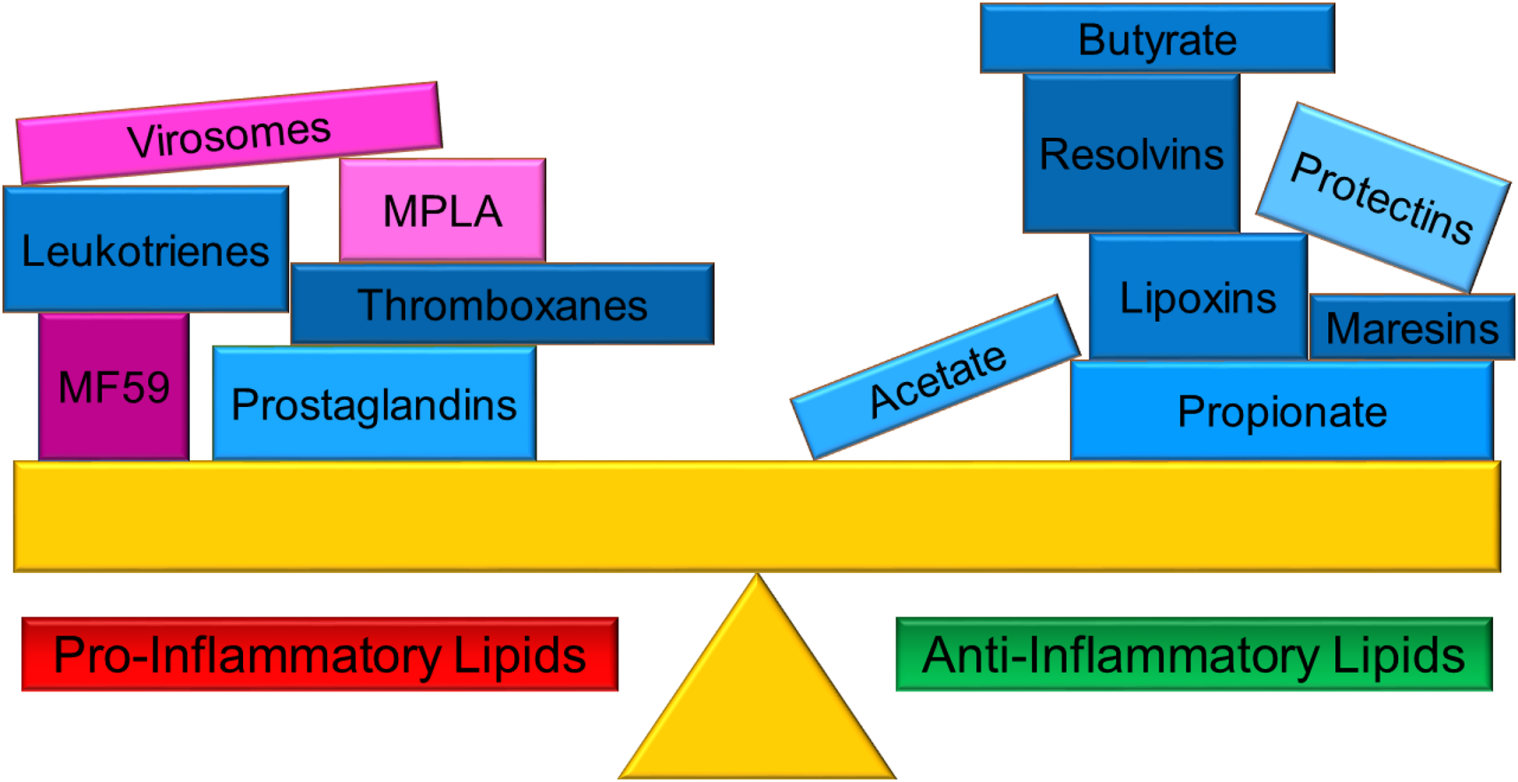
Overview of discussed immunomodulatory lipids. Immunomodulatory lipids either activate or suppress immune activation. Certain eicosanoids (metabolic products of ω‐3 and ω‐6 PUFA: prostaglandins, thromboxanes, and leukotrienes) and lipid adjuvants (virosomes, MPLA, and MF59) are pro‐inflammatory. Other eicosanoids such as lipoxins, the specialized pro‐resolving lipid mediators (metabolic products of ω‐3 PUFA; resolvins, protectins, and maresins), and SCFAs (acetate, propionate, and butyrate) are anti‐inflammatory. Natural lipids and their derivatives are shaded in blue, synthetic lipids are shaded in violet. MPLA, monophosphoryl lipid A; PUFA, polyunsaturated fatty acid; SCFAs, short‐chain fatty acids

In this review, we will first describe the structural properties of PUFAs, their derivatives, and target signaling pathways, which leads to either activation of pro‐inflammatory immune cells and inflammatory mediators or an anti‐inflammatory effect that seeks to resolve inflammation. Next, we will describe the structure of SCFA and their dual role as signaling molecules and as epigenetic modulators of immune function, which have been harnessed in treating inflammatory diseases. We will then review lipids used as adjuvants, the immunostimulatory component that enhances the protective immune response to vaccines. We conclude with an outlook on harnessing the immunoregulatory properties of lipids to develop the next generation of immunomodulating medicines (Figure 1).

## 2 POLYUNSATURATED FATTY ACIDS

**FIGURE 2:**
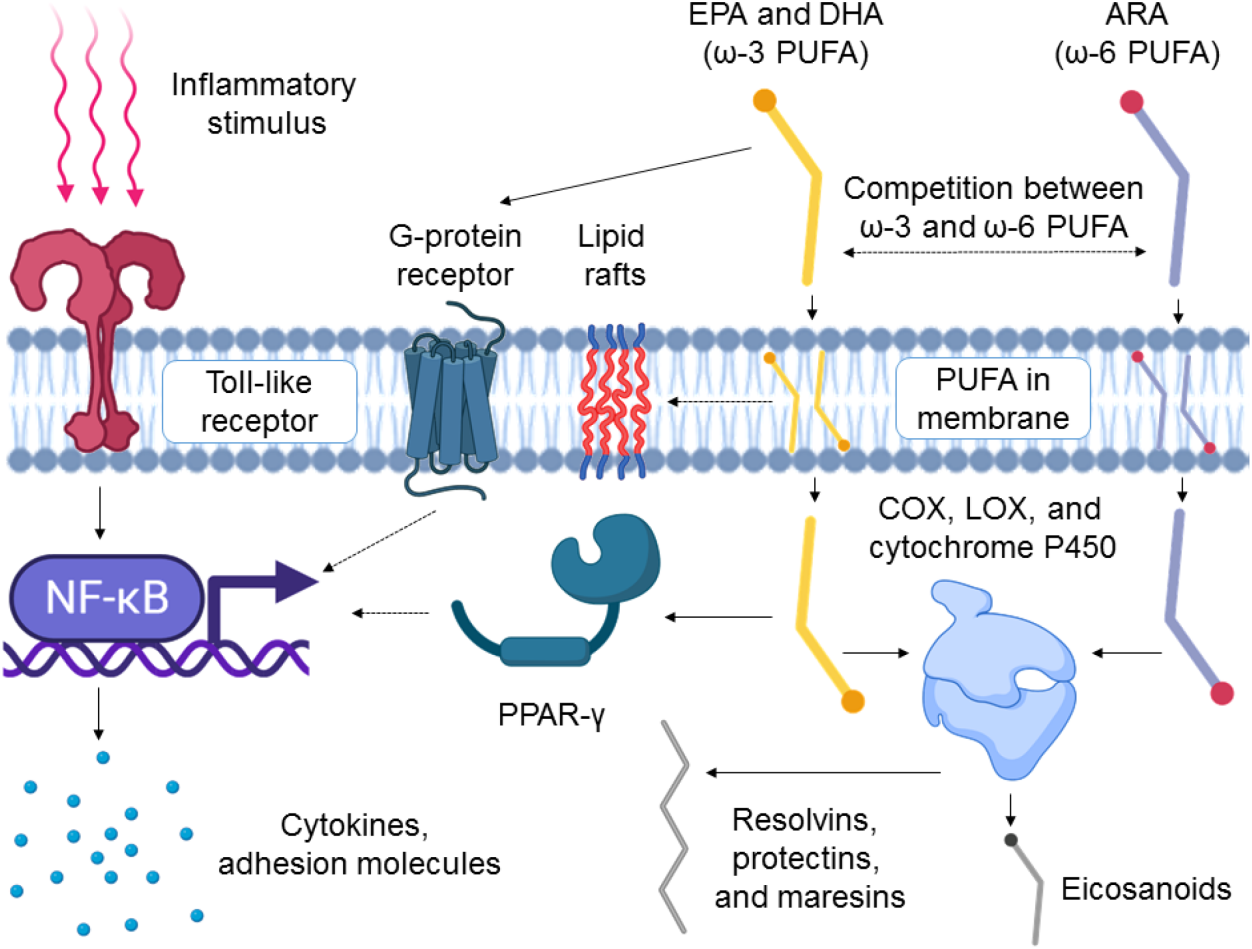
Overview of the key immunomodulatory effects of PUFAs. Free ω‐3 fatty acids (EPA and DHA) compete with free ω‐6 fatty acids (e.g. ARA) for cell membrane insertion and metabolism by COX, LOX, and Cytochrome P450 enzymes. Metabolism of ω‐3 and ω‐6 PUFA results in inflammatory or anti‐inflammatory eicosanoids respectively while metabolism of primarily ω‐3 PUFA can result in SPMs (resolvins, protectins, and maresins). ω‐3 PUFA in the cell membrane can disrupt lipid rafts housing receptors such as TLR‐4 and prevent inflammatory stimulation. ω‐3 PUFA, SPMs, and eicosanoids of ω‐3 PUFA can bind to and regulate PPAR‐γ, which subsequently binds and interferes with the translocation of NFκβ to the nucleus. ω‐3 PUFA can also act in an anti‐inflammatory manner by agonizing GPR120, which causes signal interference with the NFκβ pathway. The figure was created with BioRender.com. ARA, arachidonic acid; COX, cyclooxygenase; DHA, docosahexaenoic acid; EPA, eicosapentaenoic acid; LOX, lipoxygenase; NFκβ, nuclear factor κβ; PPAR‐γ, peroxisome proliferator activated receptor gamma; PUFAs, polyunsaturated fatty acids; SPMs, short‐chain fatty acids

The human diet comprises several types of PUFAs of which linoleic acid (ω‐6 PUFA) and α‐linoleic acid (ω‐3 PUFA) are the most common.^7^, ^8^ Linoleic acid is converted to arachidonic acid (ARA, 20:4ω‐6, pro‐inflammatory), whereas α‐linolenic acid is converted to eicosapentaenoic acid (EPA, 20:5ω‐3, anti‐inflammatory) and docosahexaenoic acid (DHA, 22:6ω‐3, anti‐inflammatory) via the same pathway, leading to enzyme competition between the two classes of PUFA.^8^ While the phospholipid composition in human immune cells can vary, in an approximate 70:20:10 mixture of T lymphocytes:B lymphocytes:monocytes, 15%–25% of the phospholipids were ARA while 0.1%–0.8% and 2%–4% were EPA and DHA, respectively.^9^, ^10^ By supplementing the diet with ω‐3 FAs, the proportion of EPA and DHA in immune cells can be elevated at the expense of ARA^8^, ^9^, ^10^ and leads to the production of less inflammatory eicosanoids (Figure 2 and Table 1).

**TABLE 1:** Major pathways and immunomodulatory effects mediated by PUFA metabolites

PUFA	Eicosanoid	Major pathway	Primarily pro‐inflammatory or anti‐inflammatory
ARA (20:4ω‐6) 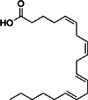	2‐series PG 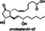	COX‐2	Pro‐inflammatory
	2‐series TX 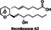	COX‐1	Pro‐inflammatory
	4‐series LT 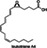	5‐LOX	Pro‐inflammatory
	4‐series LX 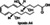	5‐LOX	Anti‐inflammatory
EPA (20:5ω‐3) 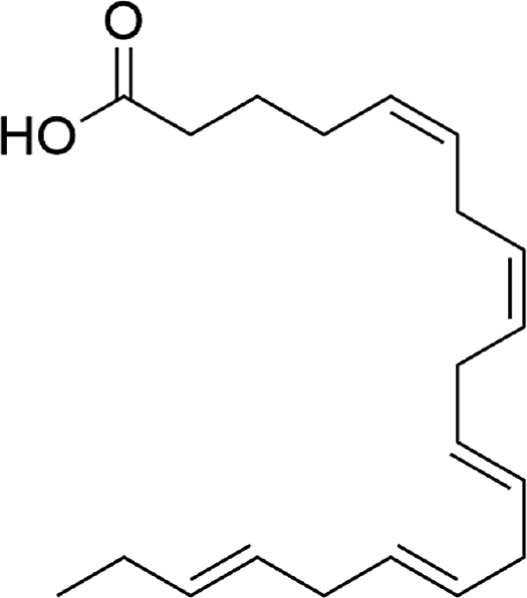	3‐series PG 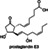	COX‐2	Weakly pro‐inflammatory
	3‐series TX 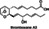	COX‐1	Weakly pro‐inflammatory
	5‐series LT 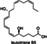	5‐LOX	Weakly pro‐inflammatory
	E‐resolvins 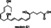	Aspirin‐acetylated COX‐2 or Cytochrome P450	Anti‐inflammatory
DHA (22:6ω‐3) 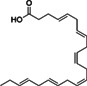	D‐resolvins 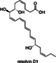	Aspirin acetylated COX‐2 or Cytochrome P450	Anti‐inflammatory
	Protectins 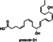	15‐LOX	Anti‐inflammatory
	Maresins 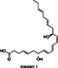	12‐LOX	Anti‐inflammatory

Abbreviations: ARA, arachidonic acid; COX, cyclooxygenase; DHA, docosahexaenoic acid; EPA, eicosapentaenoic acid; LOX, lipoxygenase; LT, leukotriene; LX, lipoxin; PG, prostaglandin; PUFAs, polyunsaturated fatty acids; TX, thromboxane.

### 2.1 Eicosanoids and inflammation

Eicosanoids are important regulators of inflammation and comprise prostaglandins (PGs), thromboxanes (TXs), leukotrienes (LTs), and lipoxins (LXs).^11^, ^12^, ^13^ These molecules are converted from PUFAs by cyclooxygenase (COX), lipoxygenase (LOX), and Cytochrome P450 enzymes. Eicosanoids released by ARA metabolism are mostly pro‐inflammatory and are the target of nonsteroidal anti‐inflammatory drugs used in suppressing inflammation.^7^ Prior to the synthesis of eicosanoids, ARA is released from the sn‐2 position of the membrane phospholipids by the action of phospholipase A_2_ enzymes, activated by inflammatory stimuli.^14^ ARA metabolism results in two‐series (two double bonds) PGs and TXs as well as four‐series (four double bonds) LTs and LXs. COX‐2 is induced in cells by inflammatory stimuli, resulting in a large increase in PG production. PGE_2_, other two‐series PGs, and the four‐series LTs are among the best‐known activators of inflammation^11^, ^12^, ^15^ and usually act through GPCRs.^16^ ω‐3 PUFAs such as EPAs are similarly metabolized by COX, LOX, and Cytochrome P450. However, EPA metabolism yields three‐series (three double bonds) PGs and TXs, and five‐series (five double bonds) LTs.^14^ By incorporating more ω‐3 PUFAs into the available pool of PUFAs, the amount of pro‐inflammatory eicosanoids is reduced by competition between ARA and the ω‐3 PUFAs.^17^ However, the metabolism of the ω‐3 PUFAs is not regulated solely by supply and demand. EPA itself can negatively regulate COX‐2 gene expression and inhibit ARA metabolism.^18^ The eicosanoids produced from metabolism of ω‐3 PUFAs such as EPAs are structurally distinct from those produced by ARA^14^ and are less biologically potent,^19^, ^20^ potentially due to a reduced receptor affinity.^21^

An important class of eicosanoids produced by the metabolism of both ω‐3 and ω‐6 PUFAs (overwhelmingly ω‐3 PUFA) are the specialized pro‐resolving lipid mediators (SPMs). This family of mediators include resolvins produced by EPA (E‐series) and DHA (D‐series) as well as protectins and maresins produced by DHA. Two series of resolvins and protectins have been identified. One series includes those derived from EPA and DHA via lipoxygenase metabolism, referred to as the S‐resolvins, S‐protectins, and S‐maresins. The second series includes those derived from aspirin‐triggered cyclooxygenase (COX‐2) or Cytochrome P450 metabolism of EPA and DHA. These lipid mediators are R‐resolvins and R‐protectins also known as aspirin‐triggered resolvins/protectins. This specialized pathway is transcellular, in which different cells start and end the metabolic pathway.^22^, ^23^, ^24^ For example, lipoxin A_4_ is a SPM that determines the extent of granulocyte accumulation and activation during inflammation. Lipoxin A_4_ formation is achieved by the transcellular biosynthesis of two sequential oxygenation reactions of ARA catalyzed by LOX in interacting cell types. One of these cells is typically a neutrophil, eosinophil, or macrophage and the other is an endothelial, epithelial, parenchymal cell, or platelet.^25^ Cell culture and animal studies have shown these metabolites to be anti‐inflammatory and inflammation resolving. For example, resolvin E1, resolvin D1, and protectin D1 all inhibited transendothelial migration of neutrophils, preventing the infiltration of neutrophils into sites of inflammation; resolvin D1 inhibited interleukin‐1β (IL‐1β) production; and protectin D1 inhibited tumor necrosis factor (TNF) and IL‐1β production.^22^, ^23^, ^24^ SPMs have been shown to influence the adaptive immune system as well. For example, D‐series resolvins and maresin 1 reduced cytokine production by activated CD8^+^ T cells and CD4^+^ T helper (Th)1 and Th17 cells while simultaneously preventing naïve CD4^+^ T‐cell differentiation into Th1 and Th17.^26^ SPMs also influence the intracellular production and extracellular release of interferon‐γ (IFN‐γ) from Th1 cells and IL‐17 from Th17 cells. Splenic T cells from mice deficient for elongase 2, the key enzyme involved in the synthesis of DHA from EPA, produced higher amounts of IFN‐γ and IL‐17 compared to cells from wild‐type control mice.^26^ By culturing naïve CD4^+^ T cells under T_reg_ differentiation in the presence of various SPM, it has been demonstrated that the de novo generation and function of FoxP3^+^ regulatory T cells (T_reg_) in the presence of D‐series resolvins and maresin 1 is enhanced.^26^ T_reg_ cultured with SPMs showed significantly enhanced FoxP3 expression, as well as increased expression of the immune checkpoint molecule CTLA‐4 and higher production of the anti‐inflammatory IL‐10 cytokine. Diet enrichment of EPA in mice,^27^ as well as humans,^28^ increased the concentration of resolvins in the blood and serum. Furthermore, resolvins reduced inflammation in a rat arthritis model.^29^ Frequent administration of the precursor of aspirin‐triggered resolvin D1 prevented joint stiffness but did not modify paw and joint edema in rats.^29^ Transgenic mice expressing *fat‐1*, a gene encoding an enzyme that converts ω‐6 to ω‐3 PUFA, showed significant improvement in a collagen induced arthritis mouse model.^30^ Clinical arthritis score, inflammatory cell infiltration, and inflammatory cytokine expression in the spleen and ankle were attenuated while T_reg_ expansion and differentiation was enhanced in *fat‐1* mice compared to wild‐type mice.

### 2.2 PUFA‐mediated suppression of pro‐inflammatory cytokines

Beyond the direct regulation of immune cells, ω‐3 PUFA derivatives downregulate the production of pro‐inflammatory cytokines such as TNF, IL‐1β, IL‐6, and IL‐8. DHA and EPA have been shown to inhibit lipopolysaccharide (LPS)‐stimulated production of IL‐6 and IL‐8 by cultured human endothelial cells,^31^, ^32^ while EPA inhibits LPS induced TNF production by cultured monocytes.^33^, ^34^ Along with reducing pro‐inflammatory cytokine production, ω‐3 PUFA derivatives can also increase the production of anti‐inflammatory cytokines. Mice fed a diet rich in EPA resulted in systemic macrophages and lymphocytes that secreted higher levels of the anti‐inflammatory cytokine IL‐10.^35^ The reduction in pro‐inflammatory cytokine production has been observed in rheumatoid arthritis patients, in whom ω‐3 PUFA dietary supplementation decreased serum TNF concentration,^36^ IL‐1 production by monocytes,^37^ and plasma IL‐1β concentrations.^38^ Reduction of systemic pro‐inflammatory cytokines correlated clinically with a decrease in the severity of inflammatory arthritis.^37^ The studies suggest that the disruption of pro‐inflammatory cytokine production by diet supplementation of ω‐3 PUFA could have a therapeutic benefit.

### 2.3 Mechanisms of PUFA‐mediated immune modulation

#### 2.3.1 Disruption of lipid rafts

The modulation of nuclear factor κβ (NFκβ), one of the main transcription factors involved in upregulation of genes encoding pro‐inflammatory cytokines, is the primary theorized mechanism by which ω‐3 PUFAs are believed to operate. EPA was shown to decrease endotoxin‐induced activation of NFκβ in human monocytes^34^, ^39^ whereas DHA has been shown to have the same effect in macrophages^40^ and dendritic cells.^41^ In contrast, saturated FA, especially lauric acid, enhanced NFκβ activation in macrophages^40^ and dendritic cells in a toll‐like receptor (TLR)‐4 dependent manner.^41^ Activated TLR‐4 and other signaling proteins associate within lipid rafts in inflammatory cells exposed to endotoxin. It was shown that DHA inhibited the ability of both endotoxin and lauric acid to promote recruitment of TLR‐4 into rafts.^42^ This observation led to one proposed mechanism that PUFAs disrupt raft formation in the membrane of inflammatory cells, thus preventing the association of signaling proteins.

#### 2.3.2 Regulation of peroxisome proliferator‐activated receptor gamma

A second proposed mechanism of PUFA influencing NFκβ activation involves the transcription factor peroxisome proliferator activated receptor gamma (PPAR)‐γ, which acts in an anti‐inflammatory manner by physically interfering with the translocation of NFκβ to the nucleus.^43^, ^44^ Eicosanoids and lipid mediators produced by the metabolism of PUFAs can bind and regulate PPAR‐γ.^45^, ^46^, ^47^ PPAR‐γ can also be activated by ω‐3 PUFAs themselves. For example, DHA activates PPAR‐γ in dendritic cells, resulting in inhibition of NFκβ activation and reduced production of inflammatory cytokines such as IL‐6 and TNF postendotoxin stimulation.^48^ In addition, DHA induces many known PPAR‐γ target genes in dendritic cells, supporting that DHA is an important anti‐inflammatory mediator.^49^

#### 2.3.3 Agonist of GPR120

A third proposed mechanism of PUFA regulation of NFκβ involves GPR120, a GPCR expressed on macrophages.^50^ ω‐3 PUFAs, agonists of GPR120, inhibited the macrophage response to endotoxin, an effect which involved maintenance of cytosolic inhibitor of nuclear factor kappa B (Iκβ) and a decrease in production of TNF and IL‐6, suggesting that GPR120 is involved in anti‐inflammatory signaling.^50^ Iκβ binds to NF‐κβ to form an inactive complex where the common pathway for NF‐κβ activation is based on phosphorylation‐induced, proteasome‐mediated degradation of Iκβ.^51^ Therefore, a maintenance of cytosolic Iκβ regulates activation of NF‐κβ. The effects produced by the synthetic agonist were similar to those produced by DHA and EPA.^50^ It was observed that both EPA and DHA, but not ARA enhanced GPR120‐mediated gene activation.^50^ Moreover, the ability of DHA to inhibit the responsiveness of macrophages to endotoxin was eliminated in GPR120 knockdown cells. These data support that the inhibitory effect of DHA on NFκβ might occur via GPR120 due to signal interference with the pathway that activates NFκβ.

## 3 SHORT‐CHAIN FATTY ACIDS

**FIGURE 3:**
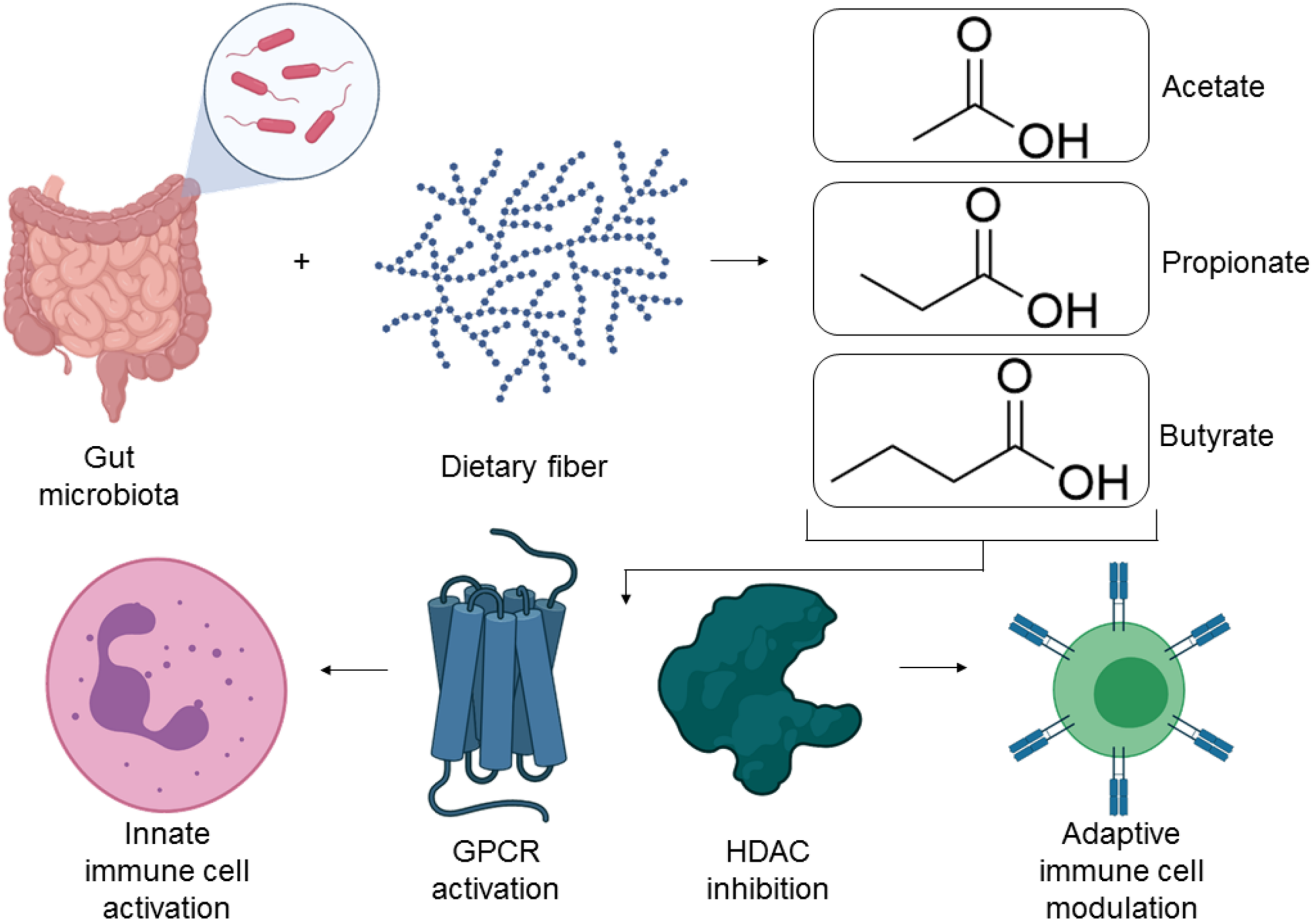
Overview of the mechanisms of SCFA‐mediated immune modulation. SCFAs are produced by anaerobic metabolism of dietary fiber in the gut by bacteria. These two‐carbon (acetate), three‐carbon (propionate), and four‐carbon (butyrate) metabolic products influence the immune system by two modes of action, GPCR activation and HDAC inhibition. SCFAs modulate the innate immune system by activating GPR41, GPR43, and GPR109a, which are expressed on cells such as monocytes, neutrophils, and macrophages. SCFAs modulate the adaptive immune system by inhibiting HDAC, leading to modulation of the mTOR pathway, subsequently modifying the ratio of effector to regulatory T cells. The figure was created with BioRender.com. GPCR, G‐protein‐coupled cell membrane receptor; HDAC, histone deacetylase; mTOR, mammalian target of rapamycin; SCFA, short‐chain fatty acids

SCFAs are carboxylic acids and are one to six carbon atoms in length. Acetate (two carbons), propionate (three carbons), and butyrate (four carbons) are the most abundant SCFAs produced by anaerobic fermentation of fiber in the digestive system and make up 90% of SCFAs in the gut.^52^ *Firmicutes* (gram‐positive) and *Bacteroidetes* (gram‐negative) are the most prolific phyla in the gut, with *Firmicutes* mostly producing butyrate while *Bacteroidetes* mainly producing acetate and propionate.^53^, ^54^ Treatment with antibiotics eliminates most SCFA producing bacteria, resulting in a significant reduction of SCFA concentration in the gut.^55^ As much as 90%–95% of SCFAs produced in the colonic lumen are absorbed by the gut mucosa^56^ and therefore the effect of SCFA is primarily on the intestinal mucosa.^6^ An important function of SCFAs is to modulate the activity of immune cells^57^ and is achieved through the activation of GPCRs and inhibition of HDAC (Figure 3).^58^, ^59^, ^60^

### 3.1 SCFAs modulate innate immune cell through GPCRs

SCFAs activate immune cell associated GPCRs and induce an anti‐inflammatory effect. GPR41 (also known as free fatty acid receptor 3 [FFAR3]), GPR43 (FFAR2), and GPR109a (hydroxycarboxylic acid receptor 2) have been identified as receptors for SCFAs and are found in the intestinal epithelial cells (IECs),^61^, ^62^ monocytes, neutrophils, and macrophages.^63^ GPR41 is primarily expressed in cells associated with adipose tissue whereas GPR43 is abundant in the aforementioned immune cells.^64^ GPR41 and GPR43 are efficiently activated by acetate, propionate, butyrate, and other SCFAs^64^ while GPR109a is activated mainly by butyrate.^65^ GPR41, GPR43, and GPR109a are coupled with G_i_ protein alpha subunit (G_i/o_) and their activation by SCFA inhibits cAMP production.^66^ Among all the SCFAs tested, propionate has the strongest agonistic response; acetate is selective for GPR43; butyrate and isobutyrate are selective for GPR41.^66^ GPCR‐deficient mice exhibit severe and uncontrolled inflammation in models of dextran sulfate sodium induced arthritis, asthma, and colitis.^67^ GPR41 is also important for facilitating the immunomodulatory effect of propionate, demonstrated in a model of allergic airway inflammation (AAI). AAI was not mitigated in GPR41^−/−^ mice treated with propionate after house dust mite exposure, but the same treatment ameliorated AAI in wild‐type and GPR43^−/−^ mice.^68^ Neutrophil‐associated GPR43 has been shown to have a role in the recruitment of polymorphonuclear leukocyte to the site of inflammation, likely in a protein kinase p38α‐dependent manner.^69^ GPR43 activation has also been shown to induce chemotaxis of neutrophils in vitro.^70^ GPR43 on IEC activates the NLR Family Pyrin Domain Containing 3 (NLRP3) inflammasome and enhances the production of IL‐18, a critical component for maintaining epithelial integrity and intestinal homeostasis.^71^ As with ω‐3 PUFA derivatives, SCFA suppress cell adhesion molecules expressed by activated monocytes, neutrophils, and endothelial cells, mitigating the infiltration of inflammatory immune cells.^72^, ^73^, ^74^ Acetate inhibits LPS‐stimulated TNF production by peripheral blood mononuclear cells in both mice and humans via GPR43.^75^ GPR43 activation has been demonstrated to induce the differentiation and enhance the function of FoxP3^+^ T_regs_ through epigenetic modulation.^76^

### 3.2 SCFA‐mediated adaptive immune cell modulation by histone deacetylase inhibition

SCFAs can modulate immune cell activity though the inhibition of histone deacetylases (HDACs), which are enzymes that remove the acetyl groups from histones, thereby tightening the chromatin structure around the DNA and repressing the activity of various transcription factors.^57^ Through HDAC inhibition, SCFAs can modulate both the effector and regulatory functions of T cells. Although it was originally thought that HDAC inhibition by SCFAs was dependent on GPR43, it has recently been shown that the absence of GPR43 does not affect HDAC inhibition by SCFAs.^59^ Rather, it was demonstrated that the specific target of acetylation in T lymphocytes is p70 s6 kinase (S6K) which can be acetylated at lysine 516 by HDAC inhibitors (e.g., SCFAs) and coactivator p300.^59^ S6K phosphorylates ribosomal protein 6 (rS6), which is an important target in the mammalian target of rapamycin (mTOR) pathway and is a key metabolic pathway in T cells. In general, mTOR activity promotes effector T cells at high levels and promotes T_reg_ at low levels. Increased phosphorylation of rS6 was observed under acetate and propionate supplementation.^77^ Therefore, SCFAs can increase mTOR activity and support the generation of both effector and T_reg_.

It has also been demonstrated that SCFA promote the function of T_regs_.^76^ SCFAs increase the activity of FoxP3^+^ T cells and IL‐10 production via HDAC inhibition, which regulates gene expression of the FoxP3 and IL‐10 loci at steady state.^78^, ^79^ SCFA can also boost the generation of Th1 and Th17 cells during active immune responses.^59^, ^80^ These observations lead Park et al. to hypothesize that SCFAs boost T‐cell responses in a manner dependent on host conditions.^80^ Under homeostatic conditions, SCFAs promote immune tolerance. However, during an active immune response, SCFAs promote the generation of an inflammatory response. Cytotoxic CD8^+^ T cells are also influenced by SCFAs, where SCFAs increase the cytotoxic activity and IL‐17 production capacity of CD8^+^ T cells.^81^ Furthermore, butyrate enhances the memory T‐cell response upon antigen rechallenge.^82^ In addition, chronic SCFA feeding has been demonstrated to induce Th17‐mediated urethritis^80^; therefore, the application of SCFAs for immunoregulation is likely context dependent.

Butyrate and propionate have been demonstrated to inhibit activation‐induced cytidine deaminase (AID) and Blimp1 expression through dose‐dependent epigenetic HDAC inhibitory activity in mouse and human B cells, which upregulates select miRNAs targeting *Aicda*‐ and *Prdm1*‐3′ UTRs.^83^ SCFA induced HDAC inhibition of B cell AID and Blimp1, inhibited class‐switch DNA recombination, somatic hypermutation, and plasma cell differentiation in T‐dependent and T‐independent antibody responses in C57BL/6J mice, T‐cell receptor (TCR)β^−/−^Tcrδ^−/−^ mice, and NOD‐*scid* IL2Rgamma^null^ (NSG) mice grafted with purified B cells.^83^ These effects were extended to autoantibody responses in lupus‐prone MRL/Fas^lpr/lpr^ and NZB/W F1 mice,^83^ supporting a potential role of SCFA in systemic lupus erythematosus therapy.

### 3.3 Therapeutic role of SCFAs in autoimmune diseases

SCFAs have been demonstrated to be immune modulating in a range of autoimmune diseases, particularly those that affect the gut. In active IBDs including Crohn's disease (CD), and ulcerative colitis (UC), SCFA‐producing bacteria (particularly those of phylum *Firmicutes*) are reduced, leading to dysbiosis. In particular, a decrease in *Firmicutes prausnitzii*, a butyrate producing bacteria from the *Clostridium* cluster IV, is an indicator of IBD.^84^, ^85^ Dysbiosis results in a decreased amount of SCFAs in the fecal matter of patients with IBD.^77^ One study found that acetate and propionate, but not butyrate, are reduced in UC patients^86^ while a separate study found both butyrate and propionate to be reduced in IBD patients.^87^ The apparent differences might be attributed to dietary differences and the method of analysis.^86^, ^87^ Directly administering SCFA enemas show clinical and histological improvement in active UC patients.^88^ Butyrate enemas also decrease NF‐κβ translocation in lamina propria macrophages in tissue sections from distal UC patients,^88^ as well as in LPS‐induced cytokine expression and NF‐κβ activation in LP mononuclear cells and PBMCs from CD patients.^89^

In a rat sepsis model, butyrate prevented liver, kidney, and lung damage, thereby improving the survival rates.^90^ The increase in survival rate was shown to be caused by downregulation of high‐mobility group box protein 1 (HMGB1) by butyrate supplementation, which is a pro‐inflammatory cytokine that activates multiple membrane receptors, including receptors for advanced glycation end products^91^ and possibly TLR‐2 and TLR‐4. Through these receptors, HMGB1 contributes to the pathogenesis of inflammatory and infectious disorders such as sepsis, arthritis, and ischemia reperfusion injury.^92^ In mouse models with acute lung injury caused by sepsis, butyrate led to a significant attenuation of liposaccharide induced damage in mice as assessed by lung histopathological changes, lower alveolar hemorrhage, and reduced neutrophil infiltration.^93^ Inhibition of HMGB1 release was postulated to be the cause of the observations. Propionate has been shown to protect from hypertensive cardiovascular damage by reducing susceptibility to cardiac ventricular arrhythmias, cardiac hypertrophy, fibrosis, and vascular dysfunction in wild‐type Naval Medical Research Institute (NMRI) and apolipoprotein E^−/−^ mice. In these mice, hypertension was induced by administration of angiotensin II and subsequently, the therapeutic effect of infusing the drinking water with propionate was tested. It was found that there was a significant reduction in both splenic effector T cells and splenic Th17 cells in both models as well as a decrease in local cardiac immune cell infiltration in wild‐type NMRI mice fed propionate compared to controls.^94^

SCFAs have the potential to exacerbate inflammation. For example, it has been documented that chronic elevation of SCFA higher than physiological levels can cause T‐cell‐induced inflammatory responses in the renal system.^80^ In addition, in mouse models of colonic inflammation, the therapeutic effect of SCFA has been inconsistent and may be context dependent. For example, SCFA enemas did not prevent or reduce intestinal damage in 2,4,6‐trinitrobenzene sulfonic‐acid (TNBS)‐induced colitis in rats^95^ but reduced colonic mucosal damage and serum inflammatory cytokines (IL‐6, TNF, and IL‐1β) in dextran sodium sulfate (DSS)‐treated mice.^96^ In contrast, butyrate did not prevent DSS‐induced intestinal damage in mice exposed to antibiotics.^97^ Similarly, butyrate was less effective in eliciting an anti‐inflammatory response in the TNBS‐induced colitis mouse model, although it induced IL‐10 and reduced IL‐12 and TNF.^98^

## 4 LIPID ADJUVANTS

**FIGURE 4:**
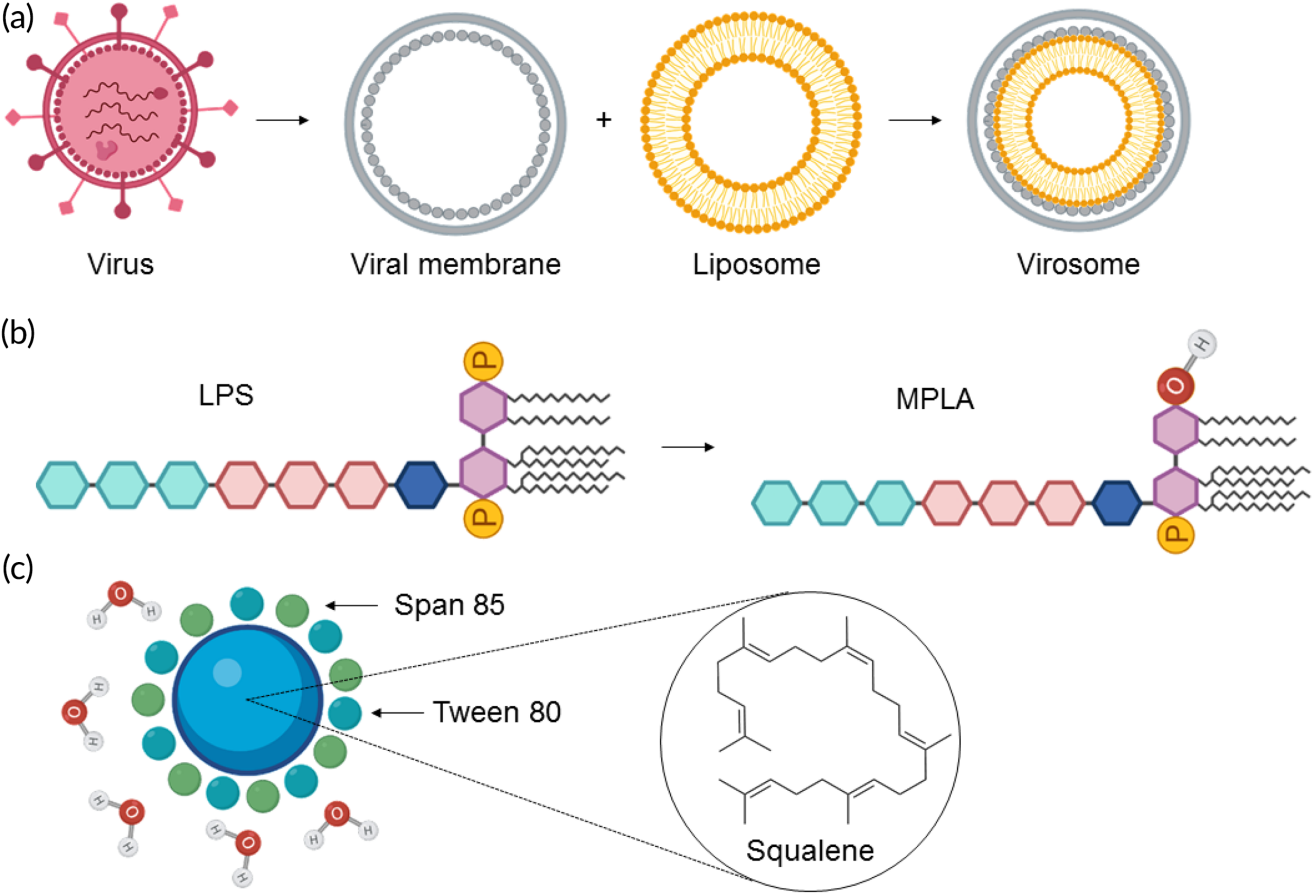
Overview of synthetic lipid adjuvants. (a) Virosomes are formed by first saponifying a virus to isolate its membrane. The virus membrane is then enveloped over a liposome containing an antigen payload. The subsequent virosome can “infect” cells and deliver the antigen in a self‐adjuvanting fashion. (b) MPLA is generated from LPS by hydrolytic cleavage of one of the phosphoryl groups to a hydroxyl group, resulting in a less toxic form of LPS with similar potent TLR‐4 stimulatory properties. (c) MF59 is an oil‐in‐water adjuvant approximately 160 nm in diameter, formulated as an emulsion of the adjuvant lipid squalene solubilized by surfactants Span 85 and Tween 80. The figure was created with BioRender.com. LPS, lipopolysaccharide; MPLA, monophosphoryl lipid A

Advances in pathogen characterization techniques and an improved understanding of the host immune response to an infection have enabled the development of subunit vaccines based on recombinant antigens containing highly purified components with excellent safety profiles. However, the immunogenicity of such defined vaccines may be lower than live attenuated or inactivated whole pathogen vaccine preparations. Therefore, subunit vaccine development often relies on adjuvants, which are molecular components that can safely boost the immunogenicity of the vaccine. Since its initial use in the 1920s, insoluble aluminum salts (alum) remained the only adjuvant in licensed vaccines for several decades.^99^ As the overall effectiveness of an immunization regimen can be improved by adjuvanting the vaccine formulation, the development of new adjuvants is an important focus in vaccines formulations.^100^ In particular, an evolving understanding of the relationship between lipids and antigen presenting cells has made it possible to design safe lipid adjuvants with strong and robust immune stimulating effects (Figure 4).

### 4.1 Free lipids as vaccine adjuvants

It is known that free cationic lipids, such as those with quaternary ammonium head groups, disrupt cell monolayers and can cause hemolysis of red blood cells and damage tissue at the injection site.^101^ However, in liposomal formulations where lipids self‐assemble into a spherical bilayer with an encapsulated aqueous core, the cationic surface has been demonstrated to promote interaction with the anionic antigen‐presenting cells (APCs) leading to a robust adaptive immune response.^102^ Cationic lipopolyamines, characterized as an unsaturated lipid tail with a long chain headgroup containing multiple amines and/or amides, were recently shown to activate both TLR‐4 and TLR‐2, resulting in strong humoral and cellular immunity in mice vaccinated against ovalbumin.^103^ The mechanism of the lipopolyamines to activate TLR‐2 and TLR‐4 is different from bacterial pathogen‐associated molecular patterns and likely linked to their fusogenic properties. Here, polyethyleneimine (PEI) has long been used as a potent cationic gene delivery system and demonstrated to have adjuvant‐like properties,^104^ but remains limited by toxicity.^105^ By attaching a lipid hydrophobic domain to low‐molecular‐weight PEI, it may be possible to generate immune stimulators with low cytotoxicity.^106^ In contrast to cationic liposomes, anionic lipids are known to be recognized by macrophages via phosphatidylserine (PS) receptors. Apoptotic cell clearance is achieved by recognition of the PS exposed on the outer leaflet of the plasma cell membrane as the cells die. By synthesizing a liposome rich in PS, the target immune system recognized the liposomes and antigens within it as self and stimulated a regulatory immune response in models of multiple sclerosis and Type 1 diabetes.^107^, ^108^ Ionizable lipids in SARS‐CoV‐2 vaccines were recently shown to have self‐adjuvating activity, supporting the role of charge in the adjuvant potential of free lipids.^109^

### 4.2 Virosomes

Virosomes are virus‐like particles, consisting of reconstituted influenza virus envelopes lacking the genetic material of the native virus, and have been explored for their enhanced immunogenic effects to improve on current influenza vaccination. By enveloping a liposome in the glycoproteins of a virus, the liposome gains the receptor‐binding membrane fusion activity of viral hemagglutinin without infecting the cells.^110^ Virosomes have been of interest to medical research as they retain the infective ability of viruses to bind and penetrate the cell, stimulate both humoral and cellular immunity, and are structurally and morphologically similar to infectious viruses. This technology has been approved in two vaccines, Epaxal® for Hepatitis A^111^, ^112^ and Inflexal® for influenza.^113^ Epaxal® is formulated with a formalin‐inactivated Hepatitis A virus that resulted in 88%–97% seroprotection 2 weeks after a single injection. After a second booster dose, Epaxal® has been found to provide robust immunity for up to 20 years.^114^ This was achieved without alum adjuvant, suggesting the adjuvant potential of virosomes. A direct comparison of Epaxal® with an alum‐based vaccine showed similar immunogenicity with fewer local reactions.^112^ Inflexal® is formulated of a haemagglutinin surface molecule of the influenza virus, which is attached to the lecithin phospholipid bilayer virosome. This formulation generates spherical vesicles ~150 nm in diameter.^115^ Despite its low viral content, Inflexal® imparts a robust immune response compared to conventional influenza vaccines.^116^, ^117^ Virosomes are also being further explored for malaria and respiratory syncytial virus vaccines.^118^, ^119^

### 4.3 Monophosphoryl lipid A

Lipid based adjuvants are currently used in clinically approved vaccines. Monophosphoryl lipid A (MPLA) is a detoxified form of the endotoxin LPS by the hydrolytic processing of LPS from *Salmonella minnesota* to a major species possessing six acyl side chains, no polysaccharide side chains, and one phosphoryl group.^120^ MPLA was tested for toxicity and immunomodulatory function by measuring the amount of MPLA needed for lethal effect in chick embryos and for protection from growth of an intradermally implanted tumor cell line in a guinea pig tumor model. The data showed that MPLA had very low toxicity compared to LPS, while functioning comparably in the tumor protection assay.^121^ While MPLA and LPS are both strong TLR‐4 agonists, MPLA's lower toxicity may be explained by biased signaling of the TLR‐4‐proximal adapter proteins, myeloid differentiation factor 88 (MyD88) and Toll‐interleukin 1 receptor domain–containing adapter inducing interferon‐β (TRIF). The low toxicity of MPLA's adjuvant function is associated with a bias toward TRIF signaling.^122^ MPLA was shown to augment antigen‐dependent T‐cell activation and expansion through mediated TRIF‐biased signaling.^122^ It has also been reported that pro‐inflammatory cytokine production by DCs in response to MPLA is dependent on both MyD88 and TRIF signaling but upregulation of DC costimulatory molecule expression is solely TRIF dependent.^123^ MPLA potently facilitated neutrophil mobilization and recruitment, a function that was largely regulated by the CXCR2 ligands CXCL1 and CXCL2, as well as the hematopoietic factor G‐CSF, and were reliant on both MyD88‐ and TRIF‐dependent signaling, although some predominance of the MyD88‐dependent pathway was evident. MPLA also induced expansion of leukocytes and their progenitors in spleen and bone marrow, as well as modulated the expression of neutrophil adhesion molecules. Those alterations were attenuated in both MyD88‐ and TRIF‐deficient mice, suggesting contributions from both pathways.^124^ Vaccines containing MPLA, such as Cervarix®, have gained approval of the United States Food and Drug Administration (FDA), making MPLA the first adjuvant since the introduction of alum 100 years ago, to be approved for use in prophylactic immunization.

### 4.4 Oil‐in‐water nanoemulsions

MF59 is a safe and effective vaccine adjuvant, formulated as an oil‐in‐water nanoemulsion of squalene that produces approximately 160‐nm‐sized droplets,^125^ originally approved for use in an influenza vaccine for the elderly in Europe in 1997 (Fluad®). In studies directly comparing MF59 to alum, MF59 was shown to be the more potent adjuvant for the induction of both antibody and CD4^+^ T‐cell responses, sparking interest into determining this adjuvant’s mode of action.^126^, ^127^ Since alum is thought to be a long‐lasting adjuvant depot, mechanistic studies assessing any similarities in MF59 were conducted at the injection site by monitoring clearance of radiolabeled antigen and squalene in rabbits.^128^ After 6 h, only 10% of the labeled squalene and 25% of the labeled antigen was at the injection site, falling to 5% and 0.05%, respectively, after 120 h. It was also found that antigen binding to the emulsion droplets is not necessary to induce the adjuvant effects. Rather, MF59 was found to create an immune responsive environment at the injection site. If MF59 was administered up to 24 h before the antigen at the same injection site, the adjuvant effects were preserved, but not vice versa.^128^ If the adjuvant and the antigen were administered to different injection sites, no adjuvant effect was observed. It was then hypothesized that MF59 adjuvant injection induces a local chemokine secretion that manifests in the recruitment of mononuclear cells from the blood. A study showed that the cellular influx observed in CCR2^+/+^ mice was significantly different than the influx found with CCR2^−/−^ mice, supporting the theory that cellular recruitment is the mode of action of MF59.^129^ Another study showed that mice deficient in ICAM‐1 showed significantly lower antibody titers against a *Plasmodium falciparum* vaccine than did wild type controls.^130^ This ICAM‐1 dependent immune response was found to be a specific mechanism for emulsion adjuvants such as MF59 but not for immune potentiator adjuvants such as MPLA. In vitro studies with MF59 found that rather than DCs being the target of this adjuvant, monocytes, macrophages, and granulocytes were targeted by MF59.^131^ Accordingly, it was postulated that a key component of the mechanism of MF59 was chemokine‐driven immune cell recruitment and chemokine‐release that would create a positive feedback loop, strongly enhancing the numbers of immune cells at the injection site. These cells could then further participate in antigen uptake and transport to the draining lymph nodes. AS03® is another squalene, oil‐in‐water emulsion adjuvant that was approved for use as an emergency pandemic adjuvant for influenza by the European Medicines Agency. AS03® was more effective than alum in inducing a high antigen‐specific antibody response and induced higher levels of cytokines and stimulated more monocyte and granulocyte recruitment to the draining lymph nodes than aluminum hydroxide.^132^

## 5 CONCLUSIONS

Lipids are important mediators of immune homeostasis and may be leveraged for treating a range of disorders. Their full potential as stand‐alone immunomodulators or adjuvants is only now beginning to be realized. In this review, we focused on three classes of lipids—two naturally occurring FAs: PUFAs and SCFAs, and adjuvant lipids for immune modulation, in which they might promote the activation or suppression of immune cell subsets in a spatially and temporally controlled manner. This effect is derived from the inherent structure of lipids, which is suited to partition and selectively accumulate in tissue microenvironments. We then summarized the mechanism, effect, and their role in disease. While the naturally occurring lipids can result in a pro‐ or anti‐inflammatory immune response, adjuvant lipids are designed to promote immune activation.

Despite showing promise, some important roadblocks must be addressed. A major limitation has been delivering PUFA/SCFA to the target location at sufficient concentrations. For example, enema‐based delivery of SCFA to the colon needs to be given frequently, multiple times a day, which render it impractical as a widespread therapy.^88^ The use of oral preparations is appealing but thus far has not been successfully used for colonic delivery. Moreover, the primary evidence of the immunomodulatory properties of SCFAs is from preclinical animal models. Besides the inherent limitation of animal models to fully recapitulate human disease, in studies using radiolabeled SCFA, it has been demonstrated that endogenous turnover of SCFAs is much lower in humans than in smaller mammals such as dogs and rats, reflecting differences in the production and utilization of SCFA between species.^133^ Such endogenous variation presents a challenge to identify a therapeutically relevant window in humans and a barrier to the extrapolation of the effects of SCFA from animal models to human disease. The limitation contributes, at least in part, to the elusiveness of consistent benefits in human interventional studies and is compounded by the lack of established methods to directly measure SCFA in vivo.^56^

The immunomodulatory effect of PUFA has been assessed solely based on diet supplementation.^17^, ^134^, ^135^ Unlike SCFA, the immune modulating properties of PUFA are not due to the PUFA themselves, but rather from the eicosanoids derived from PUFA metabolism.^11^, ^12^, ^13^ Therefore, an additional consideration is the metabolism of PUFA in target tissues. Reflecting this complexity, clinical trials using ω‐3 PUFA have garnered mixed results. For example, dietary supplementation of ω‐3 PUFA has been extensively studied in the context of cardiovascular disease.^136^, ^137^ The Reduction of Cardiovascular Events with Icosapent Ethyl‐intervention Trial (REDUCE‐IT)^138^ demonstrated the benefits of an ethyl ester form of EPA on cardiovascular disease endpoints. However, the Long‐Term Outcomes Study to Assess Statin Residual Risk with Epanova® in High Cardiovascular Risk Patients with Hypertriglyceridemia (STRENGTH),^139^ the Vitamin D and Omega‐3 Trial (VITAL),^140^ and a Study of Cardiovascular Events in Diabetes (ASCEND),^141^ did not find that ω‐3 PUFA supplementation significantly reduced major adverse cardiovascular events in high‐risk patients. While the dietary route of ω‐3 PUFA supplementation is inconclusive, other routes of drug delivery for immunomodulation may be provide more conclusive evidence.

Clinical trials using SCFAs are not as extensive as those for PUFAs, and dietary changes that influence the gut microbiome are typically studied rather than direct interventions with SCFAs. For example, the Fermented and Fiber‐rich Food (FeFiFo) study asked patients to consume either a diet richer in fiber or higher in fermented food to influence the gut microbiome and modulate the immune system in healthy adults. The FeFiFo study found that a high fiber diet did not decrease inflammation while a diet high in fermented foods did show a decrease in inflammatory markers.^142^ In studies where SCFAs are used directly as a therapeutic, the results are inconclusive. In a small study of four diversion colitis patients, treatment with an SCFA enema appeared to improve histological and endoscopy disease scores.^143^ However, this effect was less clear in randomized placebo‐controlled clinical trials in larger cohorts of patients with active distal uncreative colitis and diversion colitis, where the SCFA enema treatment did not improve clinical metrics of disease compared to a placebo.^144^, ^145^, ^146^, ^147^ As with PUFAs, other routes of drug delivery for immunomodulation may provide more conclusive evidence (Table 2).

**TABLE 2:** Clinical trials of fatty acids and lipid adjuvants

Study name	Clinicaltrials.gov ID (https://www.clinicaltrials.gov/)	Year	Intervention	Clinical trial phase	Brief summary	Primary outcome	Ref.
PUFAs							
VITAL	NCT01169259	2010–present	Daily dietary supplements of ω‐3 fatty acids (Omacor® fish oil, 1 gram)	3	Primary incidence of cancer and cardiovascular disease	Supplementation with ω‐3 fatty acids did not reduce major cardiovascular events or cancer compared with a placebo	140
ASCEND	NCT00135226	2005–present	Daily ω‐3 fatty acids (1 gram)	4	Primary incidence of cardiovascular disease in patients with diabetes	Supplementation with ω‐3 fatty acids did not reduce major cardiovascular events compared with a placebo	141
REDUCE‐IT	NCT01492361	2011–2018	Twice daily ethyl‐EPA (2 g per dose for 4 g daily)	3	Cardiovascular events in statin‐treated patients with mixed dyslipidemia	The risk of ischemic events, including cardiovascular death, was significantly lower among those who received 2 g of ethyl‐EPA twice daily than among those who received placebo	138
STRENGTH	NCT02104817	2014–2020	Daily Epanova® (ω‐3 fatty acids, 4 grams)	3	Incidence of cardiovascular disease in high‐risk patients with hypertriglyceridemia and low high‐density lipoprotein	Among statin‐treated patients at high cardiovascular risk, the addition of omega‐3 FA, compared with corn oil, to usual background therapies resulted in no significant difference in a composite outcome of major adverse cardiovascular events	139
Omega‐3 fatty acids in chronic periodontitis	NCT01997853	2012–2013	Daily ω‐3 fatty acids for 12 weeks (300 mg) or placebo	Postapproval	Therapeutic outcomes in patients with chronic periodontitis	A significant reduction in the gingival index, sulcus bleeding index, pocket depth, and clinical attachment level was found in the ω‐3 FA treatment group compared to the placebo treated group after 12‐weeks. No significant changes in serum C‐reactive protein levels	148
SCFAs							
FeFiFo study	NCT03275662	2016–2017	20 g/day increased fiber or 6 servings of fermented foods/day	N/A	General effect on inflammation, microbiota diversity, and SCFA production due to dietary changes	High‐fiber diet increased SCFA production but had no effect on cytokine response score; high‐fermented food diet decreased inflammatory markers	142
Lipid adjuvants							
Safety and immunogenicity of an adjuvanted trivalent influenza vaccine in children 6 to <72 months of age in Mexico	NCT02255279	2014–2015	Dose of trivalent influenza vaccine (aTIV) with or without MF59 adjuvant in children (6–72 months old)	3	Safety and immunogenicity of aTIV in children	aTIV was highly immunogenic and well tolerated in healthy children. Addition of adjuvant MF59 elicited a greater immune response compared to the nonadjuvanted vaccine	149
Assess the safety and immunogenicity of stored inactivated influenza H5N1 virus vaccine given with and without stored MF59 adjuvant	NCT02680002	2016–2017	2 doses of long‐term stored inactivated monovalent influenza A/Vietnam/H5N1 virus vaccine administered intramuscularly with or without MF59 adjuvant	2	Assess the safety and immunogenicity of long‐term stored vaccine	Stockpiled vaccines were well‐tolerated, adverse events were generally mild, and there was no drop in immunogenicity to the oldest stockpiled A(H5N1) vaccine. Compared to unadjuvanted vaccine, greater peak antibody responses were observed in subjects who were vaccinated with MF59‐adjuvanted vaccines, regardless of antigen dose	150
Safety and immunogenicity of MF59C.1 adjuvanted trivalent subunit influenza vaccine in elderly subjects	NCT01162122	2010–2011	Elderly patients receive one dose of aTIV (adjuvanted) or TIV (nonadjuvanted)	3	Safety and immunogenicity of aTIV in the elderly	aTIV was not only noninferior to TIV but also elicited significantly higher antibody responses at Day 22 than TIV against all homologous and heterologous strains, even in subjects with co‐morbidities. Reactogenicity was higher in the aTIV group, but reactions were mild to moderate and transient	151
Human papilloma virus (HPV) vaccine efficacy trial against cervical precancer in young adults with GlaxoSmithKline (GSK) biologicals HPV‐16/18	NCT00122681	2004–2009	Three doses of HPV‐16/18 AS04‐adjuvanted vaccine over the course of 6 months	3	Vaccine safety and efficacy against HPV in young women	The HPV‐16/18 AS04‐adjuvanted vaccine showed high efficacy against CIN2+ associated with HPV‐16/18 and nonvaccine oncogenic HPV types	152
Hepatitis A vaccine in patients with immunomodulating drugs	NCT01360970	2009–2011	Two doses of either Havrix® (alum adjuvanted) or Epaxal® (virosome adjuvanted)	2	Assess the hepatitis A virus antibody response in patients with rheumatoid arthritis treated with tumor necrosis factor‐inhibitors and/or methotrexate	Two doses of hepatitis A vaccine at a 6‐month interval provided protection for most immunosuppressed RA patients. However, a single dose did not sufficiently protect this group of patients.	153
Phase Ib trial of two‐virosome formulated malaria vaccine components (PEV 301, PEV 302) in Tanzania (PMAL03)	NCT00513669	2008–2009	Two doses of virosome formulated antimalaria vaccine components (PEV301 and PEV302) compared to two doses of virosome influenza vaccine (Inflexal®)	1	Assess safety and immunogenicity of two virosome formulated antimalaria vaccine components (PEV 301 and PEV 302) administered in combination to healthy semi‐immune adults and children (i.e,. an individual infected by *Plasmodium falciparum* who is asymptomatic)	Significant reduction in malaria incidence with no significant adverse effects	154
Safety and immunogenicity of a pediatric dose of virosomal hepatitis A vaccine	NCT01405677	2004–2012	Two doses of virosome adjuvanted Epaxal® Junior compared to standard dose of Epaxal® and alum adjuvanted Havrix® Junior	2	Confirm that the equivalency between a pediatric dose of Epaxal® vaccine to the standard dose against hepatitis A	Vaccination of children with two doses of Epaxal® Junior confers protection of at least 5.5 years	155

Abbreviations: ASCEND, A Study of Cardiovascular Events iN Diabetes; aTIV, adjuvanted trivalent influenza vaccine; FeFiFo: Fermented and Fiber‐rich Food; GSK, GlaxoSmithKline; HPV, human papilloma virus; REDUCE‐IT, Reduction of Cardiovascular Events with Icosapent Ethyl‐intervention Trial; STRENGTH, Study to Assess STatin Residual Risk Reduction With EpaNova® in HiGh CV Risk PatienTs With Hypertriglyceridemia; VITAL, Vitamin D and Omega‐3 Trial.

Lipid adjuvants have greatly enhanced prophylactic vaccination by inducing robust immune responses against pathogens. However, lipid adjuvants are primarily immune activating. Recent examples of engineered lipid biomaterials that mimic apoptotic cells to deliver antigens in a tolerizing fashion,^107^, ^108^ support that further development of engineered lipids could yield agents with superior therapeutic potential to that achievable with more traditional agents.

Lipids are intrinsically linked to the maintenance of immunological homeostasis and are a potent weapon in the pharmacological armamentarium for numerous inflammatory disorders (Table 3). The elucidation of the mechanistic basis of their function and of novel targeted approaches for delivery represents a desirable strategy that might achieve consistent and reliable outcomes. These may also be combined with dietary supplementation or other pharmacological interventions to maximize the therapeutic benefit in a wide range of intestinal, metabolic, and inflammatory diseases.

**TABLE 3:** Recent applications of immunomodulatory lipids in disease contexts

Lipid	Disease model	Experimental design	Observed effect	Ref.
Cancer				
DHA (ω‐3 PUFA)	Murine postmenopausal breast cancer	Ovariectomized, immune‐competent female mice orthotopically injected with Py230 mammary tumor cells fed high fat diets with or without DHA	DHA diet reduced inflammation in the obese mammary fat pad in the absence of tumor cells and inhibited Py230 tumor growth in vivo	156
ω‐3/ω‐6 PUFA	Murine NASH‐tumor model	High fat diets with differing ratios of ω‐6 and ω‐3 PUFAs were fed to streptozotocin/high‐fat diet (STZ/HFD)‐treated mice to analyze NAFLD‐related liver fibrosis and tumorigenesis	In 20‐week‐old mice, ω‐3 PUFA‐rich diets alleviated tumor load significantly, with reduced liver/body weight index, tumor size, and tumor number accompanied by significant increase in survival	157
ω‐6 PUFA	Murine mammary cancer	High‐fat (ω‐6) diets were fed to pregnant mice and mammary tumor incidence induced by 7,12‐dimethylbenz[a]anthracene in offspring	Maternal high‐fat diet intake during pregnancy induces a transgenerational increase in offspring mammary cancer risk in mice	158
DHA (ω‐3 PUFA)	Murine ovarian cancer patent derived xenograft	PDX OVXF‐550 model was fed either a control (0% DHA) or DHA (3% w/w DHA) diet and treated with or without carboplatin and tumor growth analyzed	DHA supplementation reduced cancer cell growth and enhanced the efficacy of carboplatin in preclinical models of ovarian cancer through increased apoptosis and necrosis	159
Prostaglandin E_3_ (EPA‐derived eicosanoid)	Murine PC3 xenograft model of prostate cancer	PGE_3_ or saline was injected next to the tumor every 3 days for 4 weeks before tumor excision and analysis	PGE_3_ inhibited prostate cancer in vivo in immunodeficient (nude) neoplastic mice	160
DHA (ω‐3 PUFA)	In vitro (YAMC and IMCE mouse colonic cell lines) and in vivo (*Drosophila*, wild type and *fat‐1* mice) models	Cellular DHA enrichment via therapeutic nanoparticle delivery, endogenous synthesis, or dietary supplementation and analysis of EGFR‐nanoclustering by super‐resolution microscopy	DHA enrichment reduced EGFR‐mediated cell proliferation and downstream Ras/ERK signaling	161
Butyrate (SCFA)	Murine models of colon carcinoma (MC38 and CT26) and fibrosarcoma (MCA101_OVA_)	Mice were fed diets supplemented with butyrate before inoculation with cancer cells. Mice were treated with anti‐CTLA‐4 antibodies and progression of cancer was analyzed	Systemic butyrate appeared to limit antitumor activity of anti‐CTLA‐4	162
Butyrate (SCFA, produced by *Clostridium butyricum*)	Murine model of colorectal cancer induced by high‐fat diet in *Apc* ^min/+^ mice	Mice were purged of gut bacteria by antibiotics before culturing *C. butyricum* by diet supplementation three times a week. Mice were fed either a high‐fat diet or a basal diet for 12 weeks before euthanasia to determine extent and progression of colorectal cancer	*C. butyricum* significantly inhibited high‐fat diet induced intestinal tumor development in *Apc* ^min/+^ mice. Moreover, intestinal tumor cells treated with *C. butyricum* exhibited decreased proliferation and increased apoptosis	163
Autoimmune diseases				
Pinolenic acid (ω‐6 PUFA)	PBMCs isolated from blood of rheumatoid arthritis patients	Investigated the transcriptomic profile of pinolenic treatment on LPS activated PBMCs isolated from healthy controls and RA patients	Pinolenic acid had significant effects on the regulation of metabolic and inflammatory pathways (IL‐6, TNF‐α, IL‐1β, and PGE_2_) in PBMCs from RA patients and HCs, and therefore may have beneficial anti‐inflammatory effects in patients with RA	164
Resolvin E1 (EPA‐derived eicosanoid)	Murine model of psoriasis induced by imiquimod	30 min before imiquimod administration, vehicle or resolvin E1 were administered intravenously. Ear thickness was measured 24 h after imiquimod treatment	Resolvin E1 significantly impaired imiquimod induced psoriatic dermatitis	165
Propionate (SCFA, induced by resistant starch high‐fiber diet)	Murine model of collagen induced arthritis	Mice were fed either a normal diet or a high‐fiber diet with resistant starch. β‐acids were added to eliminate SCFAs	RS‐HFD significantly reduced arthritis severity and bone erosion in CIA mice and correlated with splenic T_reg_ expansion and increase in serum IL‐10. Addition of β‐acids significantly reduced serum propionate and eliminated RS‐HFD‐induced disease improvement	166
Butyrate (SCFA)	Murine model of autoimmune hepatitis	Hepatitis was induced in mice by S100 injection. Mice were then split into control, high‐fiber diet, and sodium butyrate enriched diet groups	Mice fed with either high‐fiber diet or sodium butyrate showed significantly reduced serum aminotransferases and minor liver injury compared to mice fed with the control diet. Moreover, the ratio of T_reg_/Th17 was significantly higher in high‐fiber diet and sodium butyrate‐fed mice than the control group	167
Various SCFAs	Murine model of autoimmune experimental uveitis	Mice were immunized with an emulsion containing IRBP1–20 or IRBP651–670 and incomplete Freund’s adjuvant to induce uveitis. Mice were then fed differing SCFAs in the drinking water for three weeks before uveitis induction and during the study	Exogenous administration of SCFAs stabilized subclinical intestinal alterations that occur in inflammatory diseases including uveitis, as well as prevented the trafficking of leukocytes between the gastrointestinal tract and extra‐intestinal tissues	168
Butyrate (SCFA)	Murine model of dysregulated bile acid synthesis leading to hepatitis	FXR KO mice were fed a western diet with or without butyrate supplementation	Reduced butyrate contributes to the development of hepatitis in the FXR KO mouse model. Butyrate supplementation reverses dysregulated BA synthesis and its associated hepatitis.	169
DHA (ω‐3 PUFA)	Murine model of systemic lupus erythematosus	Lupus was induced in female NZBWF1 mice by 4 weekly intranasal instillations with 1 mg cSiO_2_. One week after the final instillation, which marks onset of ELS formation, mice were fed diets supplemented with 0, 4, or 10 g/kg DHA	Dietary intervention with high but not low DHA after cSiO_2_ treatment suppressed or delayed: (i) recruitment of T and B cells to the lung, (ii) development of pulmonary ELS, (iii) elevation of a wide spectrum of plasma autoantibodies associated with lupus and other autoimmune diseases, (iv) initiation and progression of glomerulonephritis, and (v) onset of the moribund state	170

Abbreviations: BA, bile acid; CIA, collagen‐inducted arthritis; DHA, docosahexaenoic acid; EGFR, epidermal growth factor receptor; ELS, extralobar pulmonary sequestration; EPA, eicosapentaenoic acid; FXR KO: farnesoid × receptor knock out; HCs: healthy controls; HFD: high‐fat diet; IMCE, immortomouse × MIN colonic epithelium; LPS, lipopolysaccharide; NASH, nonalcoholic steatohepatitis; NAFLD, nonalcoholic fatty liver disease; PBMC, peripheral blood mononuclear cell; PDX, patient‐derived xenograft; PGE_3_, prostaglandin E3; PUFA, polyunsaturated fatty acid; RA, rheumatoid arthritis; RS‐HFD, resistant starch high‐fat diet; SCFA, short‐chain fatty acid; STZ, streptozotocin; YAMC, young adult mouse colonic epithelium.

## CONFLICT OF INTEREST

The authors declare no conflict of interest.

## AUTHOR CONTRIBUTIONS

**Wade T. Johnson:** Conceptualization (lead); writing—original draft (lead); writing—review and editing (lead). **Nicholas C. Dorn:** Conceptualization (supporting); writing—original draft (supporting); writing—review and editing (supporting). **Dora A. Ogbonna:** Conceptualization (supporting); writing—original draft (supporting); writing—review and editing (supporting). **Nunzio Bottini:** Funding acquisition (supporting); supervision (supporting); writing—review and editing (supporting). **Nisarg J. Shah:** Conceptualization (equal); funding acquisition (lead); supervision (lead); writing—original draft (supporting); writing—review and editing (supporting).

## Data Availability

Data sharing is not applicable to this article as no new data were created or analyzed in this study.
